# One plus one equals three (or more …): combining the assessment of movement behavior and subjective states in everyday life

**DOI:** 10.3389/fpsyg.2013.00216

**Published:** 2013-05-16

**Authors:** Johannes B. Bussmann

**Affiliations:** Rehabilitation Medicine and Physical Therapy, Erasmus MC University Medical CenterRotterdam, Netherlands

Movement behavior, i.e., the overt permanence of postures and motions in daily life, is of crucial importance in human functioning. Movement behavior contributes to participating in daily life, and improves quality of life (Buffart et al., 2009). In addition, movement behavior—mostly described in terms of levels of physical activity, active lifestyle, and/or sedentary lifestyle—has shown to be related to health outcomes, such as incidence of and mortality due to diseases such as cancer, diabetes, and cardiovascular disease (United States Department of Health and Human Services, 2000; Pedersen and Saltin, 2006).

Another important aspect of movement behavior is its relationship with mood, affective states, and mental functioning. However, this relationship is quite complex and surely not uni-directional. It is generally accepted that being physically active contributes to a better mood, while affective states will influence movement behavior, in both quantitative (e.g., amount of walking) and qualitative (e.g., walking pattern) outcomes. Therefore, it seems quite obvious that much research focusing on understanding movement behavior includes mental states, while much research on understanding mental states includes movement behavior. Surprisingly, this it not the case.

Despite the important mutual relationship, linking movement behavior and mental states has not been common in the past. One of the reasons might be the separated areas of expertise—people working in the field of mental states not familiar with measurement of movement behavior and vice versa. An expression of this is the existence of two separate meetings: the International Conference of Physical Activity and Movement (ICAMPAM; next conference in Amherst, June 17–19, 2013) that is mainly attended by the “activity” researchers, and the Society of Ambulatory Assessment conference (SAA, next conference in Amsterdam, June 20–22, 2013), mainly visited by “electronic diary” researchers.

Developments in this field of research is strongly driven by technological progress. For example, within the field of activity monitoring it has resulted in several devices that measure physical activity for a prolonged period, without significant load for the participant, and quite low-cost (Bassett, 2012). Parallel to this, accelerometers and GPS nowadays are regular components of smartphones that, supported by many apps, provide data on some aspects of movement behavior. In the area of mood and mental states research considerable technological progress has been made too. Started with paper diaries, measurement of mood and mental states evoluted to e-diaries on Personal Digital Assistant (PDA)-based devices such as the Palm. Although sometimes a link was made with movement behavior, measurement of both concepts was not integrated. Also in the area of e-diaries and e-questionnaires significant progress was made with the development and more regular use of smartphones.

The use of e-diaries/questionnaires is not only relevant from the perspective of combining movement behavior and mental states or mood. The technique of e-diaries and questionnaires can also be used to get “context” information, including the situational (e.g., work, leisure time, study), social (presence of friends, colleagues, family), or geographical (the location in which the subject is) context. E-diaries and questionnaires can also be used to assess—especially in patient groups—symptoms such as pain and fatigue. Here again, a complex and mostly unsolved relationship exists between movement behavior on the one hand, and these symptoms on the other. Similar to the research are of mental states, simultaneous measurement of these constructs will contribute to a better understanding of these relationships, and to a better diagnosis and treatment of many patients.

An important future challenge in the area of combining activity monitoring with ambulatory assessment of mood and other mental states will be the application of feedback, provided directly or later on. For example, in research specific characteristics of movement behavior (or characteristics related to it, such as heart rate) can automatically initiate an assessment of mental state (interactive ambulatory assessment). Feedback can also be used in interventions and therapy: ambulatory assessment interventions. In the field of movement behavior feedback on the e.g., the number of steps has shown to be effective in improving levels of physical activity. Similarly, tailored feedback on level of physical activity can be used in therapies aimed at improving mental health.

The current research topic “Ecological Momentary Assessment and Intervention in physical activity and well-being: Affective reactions, social-cognitive factors, and behaviors as determinants of physical activity and exercise (edited by W. Schlicht, U. Ebner-Priemer, and Martina Kanning)” published in Frontiers in Movement Science and Sport Psychology nicely represent the issues discussed above and give insight in the current advances (Kanning et al., 2013). Several papers focus on measuring movement behavior (e.g., in terms of physical activity and sedentary lifestyle) and affective states, and on their mutual relationships (Dunton et al., 2012; Bossmann et al., 2013; Kanning, 2013; Von Haaren et al., 2013). But also some other areas—interactive ambulatory assessment (Ebner-Priemer et al., 2013), assessment of clinical symptoms (Murphy et al., 2012), and ways to optimize movement behavior (Schwerdtfeger et al., 2012)—are well-covered.

It can be concluded that the time is right to combine advanced methods of measuring movement behavior with advanced use of e-diaries/e-question-naires. The effect of this will not just be the result of adding the values of both separate fields, but will surpass this sum. Although the idea of combining them is not new, technological progress has resulted in products and possibilities that allow a significant next step. That next step surely includes the application of e-diaries/questionnaires focusing on mood and mental states, but is also relevant for constructs such as context, and symptoms as pain and fatigue. Combining methods will contribute to new knowledge, relevant from a fundamental scientific perspective, but also from a more clinical and treatment-based perspective. Although the first next steps will be made within research, the moment of application in individual people with individual problems or questions has moved into the foreseeable future.

## References

[B1] Bassett D. R. (2012). Device-based monitoring in physical activity and public health research. Physiol. Meas. 33, 1769–1783 10.1088/0967-3334/33/11/1769 23110847

[B2] Bossmann T. Kanning M. Koudela S. Hey S. Ebner-Priemer U. W. (2013). The association between short periods of everyday life activities and affective states: a replication study using ambulatory assessment. Front. Psychol. 4:102 10.3389/fpsyg.2013.00102 23596426 PMC3625722

[B2a] Buffart L. M. van den Berg-Emons R. J. van Meeteren J. Stam H. J. Roebroeck M. E. (2009). Lifestyle, participation, and health-related quality of life in adolescents and young adults with myelomeningocele. Dev. Med. Child Neurol. 51, 886–894 10.1111/j.1469-8749.2009.03293.x 19416327

[B3] Dunton G. F. Liao Y. Kawabata K. Intille S. (2012). Momentary assessment of adults' physical activity and sedentary behavior: feasibility and validity. Front. Psychol. 3:260 10.3389/fpsyg.2012.00260 22866046 PMC3408114

[B4] Ebner-Priemer U. Koudela S. Mutz G. Kanning M. (2013). Interactive multimodal ambulatory monitoring to investigate the association between physical activity and mood. Front. Psychol. 3:596 10.3389/fpsyg.2012.00596 23355828 PMC3554220

[B5] Kanning M. (2013). Using objective, real-time measures to investigate the effect of actual physical activity on affective states in everyday life differentiating the contexts of working and leisure time in a sample with students. Front. Psychol. 3:602 10.3389/fpsyg.2012.00602 23346064 PMC3549542

[B6] Kanning M. Ebner-Priemer U. W. Schlicht W. (2013). How to investigate within-subject associations between physical activity and momentary affective states in everyday life: a position statement based on a literature overview. Front. Psychol. 4:187 10.3389/fpsyg.2013.00187 23641221 PMC3638123

[B7] Murphy S. Kratz A. L. Williams D. A. Geisser M. E. (2012). The association between symptoms, pain coping strategies, and physical activity among people with symptomatic knee and hip osteoarthritis. Front. Psychol. 3:326 10.3389/fpsyg.2012.00326 22969747 PMC3432514

[B8] Pedersen B. K. Saltin B. (2006). Evidence for prescribing exercise as therapy in chronic disease. Scand. J. Med. Sci. Sports 16(Suppl. 1), 3–63 10.1111/j.1600-0838.2006.00520.x 16451303

[B9] Schwerdtfeger A. Schmitz C. Warken M. (2012). Using text messages to bridge the intention-behavior gap? A pilot study on the use of text message reminders to increase objectively assessed physical activity in daily life. Front. Psychol. 3:270 10.3389/fpsyg.2012.00270 22876237 PMC3410409

[B10] United States Department of Health Human Services. (2000). Healthy People 2010: Understanding and Improving Health. 2nd Edn Washington, DC: US Government Printing Office

[B11] Von Haaren B. Loeffler S. N. Haertel S. Anastasopoulou P. Stumpp S. Hey S. (2013). Characteristics of the activity-affect association in inactive people: an ambulatory assessment study in daily life. Front. Psychol. 4:163 10.3389/fpsyg.2013.00163 23580167 PMC3619104

